# Muscle size of individual hip extensors in sprint runners: Its relation to spatiotemporal variables and sprint velocity during maximal velocity sprinting

**DOI:** 10.1371/journal.pone.0249670

**Published:** 2021-04-05

**Authors:** Katsuki Takahashi, Kiyotaka Kamibayashi, Taku Wakahara

**Affiliations:** 1 Graduate School of Health and Sports Science, Doshisha University, Kyoto, Japan; 2 Faculty of Health and Sports Science, Doshisha University, Kyoto, Japan; 3 Human Performance Laboratory, Waseda University, Saitama, Japan; Universite de Nantes, FRANCE

## Abstract

Hip extensor muscle size is related to sprint running performance. However, the mechanisms underlying this relationship remain unclear. To gain insights into this issue, the present study examined the relationships between the individual hip extensor sizes, spatiotemporal variables (step frequency and length, and their determinants), and sprint velocity during maximal velocity sprinting. Magnetic resonance images of the hip and right thigh were obtained from 26 male sprinters to determine the volumes of the gluteus maximus, individual hamstrings and adductors, and gracilis. Muscle volumes were normalized to their respective body mass and recorded as relative muscle volumes. The sprinters performed a 100-m sprint with their maximal effort. Their sprint motions were recorded using cameras to calculate the mean sprint velocity and the spatiotemporal variables at 50–60 m interval. The sprint velocity was significantly correlated with the relative volume of the semitendinosus (*r* = 0.497, *P* = 0.010), but not with the volumes of the other examined muscles. The relative volume of semitendinosus significantly correlated with the stance distance (*r* = 0.414, *P* = 0.036) and the stance distance adjusted by the stance time (*r* = 0.490, *P* = 0.011). Moreover, there were significant correlations between the stance distance and step length (*r* = 0.592, *P* = 0.001), and between the step length and sprint velocity (*r* = 0.509, *P* = 0.008). These results suggest that the semitendinosus contributes to attaining long stance distance and thereby high sprint velocity during maximal velocity sprinting.

## Introduction

Sprint running is one of the fastest forms of unaided locomotion for humans on land and is a fundamental movement required in a number of sports. Many studies [1,2] have focused on the factors influencing sprint performance. Sprint velocity is the product of step frequency and step length, which are determined by the times and distances of the stance and flight phases [2]. To achieve either or both high step frequency and/or long step length, the lower limb muscles play significant roles by generating high forces during sprinting [3,4]. Of all muscles, the hip extensors, which include the gluteus maximus (GM), thigh adductors, and hamstrings, were reported to be highly activated [5,6], and exerted substantial force during both flight and stance phases [4,7], contributed to back-swing velocities of the legs [4], and produced horizontal ground reaction forces [8]. Based on these findings, the force generated by the hip extensors could be a crucial factor in sprint performance.

Since the force-generating capacity of a muscle is primarily determined by muscle size [9,10], it is easy to conjecture that having large hip extensors is advantageous for achieving high performance in maximal velocity sprinting. However, large muscles entail large body mass, having a negative effect on the body velocity. Increasing size of marginally advantageous muscles could thus impair sprint velocity [11]. Therefore, it is speculated that size of muscle(s) which contribute greatly to sprinting is related to sprint performance, whereas that of the other hip extensors is not. In this regard, several studies demonstrated that the sizes of GM [12,13], thigh adductors [14–16], and hamstrings [12,15], relative to body mass were significantly correlated with sprint velocities (or times). However, previous studies [12,15] evaluated the size of hamstring muscle group as a whole, although the group is actually comprised of three muscles (but delineated by four names: the semitendinosus [ST], the semimembranosus [SM], the biceps femoris long head [BFlh], and the biceps femoris short head [BFsh]). These muscles reportedly exhibited diverse morphologies [17] and activations during sprinting [6]. Therefore, the contributions of the individual hamstring muscles to sprint performance may be variable among them. For example, ST volume was larger in sprinters than in non-sprinters [11,13], and the magnitude of the difference (54%) was substantially greater than those in the other hamstrings (20–26%) [11]. Furthermore, mechanisms underlying the relationship between the hip extensor size and sprint velocity remain unknown. Since the sprint velocity is determined by spatiotemporal variables (step frequency and length, and their determinants) [2], clarifying the relationships among the muscle sizes, spatiotemporal variables, and sprint velocity would provide useful insights into this issue as well as valuable information to athletes and coaches for developing effective training programs according to their target spatiotemporal variables.

The purpose of the present study was to examine the relationships between the sizes of individual hip extensors, the spatiotemporal variables, and sprint velocity during maximal velocity sprinting. It was reported that GM volume was correlated with sprint time [12,13]. Among the hamstrings, the ST displayed the longest fiber length [17], which is a determinant of the maximal shortening velocity of the muscle [18] and affects the force-velocity relationship [19]. It is therefore likely that ST is more suited to generating a large force during sprinting, in which the angular velocity of the hip extension is substantially high [20]. Additionally, the activity level of ST was reported to be higher than that of BFlh in the middle flight and late stance phases during maximal velocity sprinting [6]. Considering these points, we hypothesized that GM and ST volumes relative to body mass are correlated with specific spatiotemporal variable(s) and therefore the sprint velocity.

## Methods

### Participants

Twenty-six male sprinters (age: 20.2 ± 1.2 years; body height: 1.741 ± 0.057 m; body mass: 65.8 ± 6.2 kg; mean ± standard deviation) participated in the present study. A *priori* power analysis was conducted using G*Power (version 3.1.9.2) [21] to detect statistically significant correlations between muscle volumes and sprint performances with an alpha level of 0.05 and a power of 80%. Effect size was assumed to be 0.62 based on the results of a previous study [22] that reported a significant correlation between the lower limb muscle volume and maximal sprint velocity. As a result of the power analysis, the required sample size was estimated to be 18. To account for possible attrition and missing data, 26 sprinters were therefore recruited for the present study. The sprinters engaged in sprint events (i.e., 100-, 200- and 400-m sprints), and their personal best records for the 100-m race ranged from 10.35 to 11.33 s (10.77 ± 0.27 s). They had ≥ 3 years (8.1 ± 2.7 years) of experience in sprint running. Nine of the sprinters had a history of strain injury in the hip or thigh muscles during the last 1–4 years prior to this study. All participants were informed of the purpose and potential risks of the study and provided written informed consent. The present study was approved by the Doshisha University Research Ethics Review Committee (16035) and was conducted in accordance with the Declaration of Helsinki.

### Magnetic resonance (MR) imaging

Using a 1.5-T scanner system (Echelon VEGA, Hitachi, JPN), T1-weighted MR images of the hips and right thigh were obtained. The participants were instructed to refrain from intensive practice and training on the day of the test until MR imaging was conducted. The following imaging parameters were used for scanning images: slice thickness, 0.6 cm; gap, 0.4 cm; echo time, 8.8 ms; repetition time, 500 ms; field of view, 25.6/40 cm; and acquisition matrix, 256 × 192 (reconstructed matrix, 512 × 512). The participants lay prone with their knee and hip joints extended in the magnet bore. The hip scans were conducted using a 16-channel body array coil while participants held their breath for 20-s in order to prevent the potential influence of motion artifacts resulting from respiration. To reduce the effect of fluid shift caused by the change in posture on cross-sectional areas (CSAs) of the thigh muscles [23], the participants were placed in the prone position for at least 20 min prior to the thigh scans.

The muscle CSAs of the following nine muscles were analyzed from the origins to the insertions using 3D slicer software (www.slicer.org) [24] by tracing their outlines: 1) GM, 2) ST, 3) SM, 4) BFlh, 5) BFsh, 6) adductor longus (ADL), 7) adductor brevis (ADB), 8) adductor magnus (ADM), and 9) gracilis (Gra). The volume of each muscle was calculated by summing the CSAs of each image times 1 cm (the sum of the slice thickness and interslice gap). The above analyses were performed twice by an investigator, and the mean values were used for subsequent analyses. The coefficient of variation (CV) for the two measurements was 3.6 ± 3.1%, and the intraclass correlation coefficient (ICC) was ≥ 0.911, except for ADB (ICC = 0.541) because of its unclear outlines in the scanned images. Muscle volumes were normalized to body mass and recorded as relative muscle volumes (cm^3^/kg).

### Sprint running

After a sufficient warm-up period, the participants performed a 100-m sprint with their maximal effort on a synthetic track. During the testing session, they wore their own sprint spikes. Using starting blocks, they started the sprint at arbitrary times of their own choosing. Their sprints were recorded using three cameras. Two of the cameras (EX-100PRO, CASIO, JPN, frame rate: 120 fps, exposure time: 1/2000 s) were used for panning and placed 25- and 75-m from the starting line to cover the first (0–50 m) and second (50–100 m) halves of the sprint, respectively. The third camera (HAS-U2, DITECT, JPN, frame rate: 250 fps, exposure time: 1/2000 s) was fixed on the 55 m point from the starting line to cover the 50–60 m interval, which corresponds to the maximal velocity phase of sprinting [25]. The maximal sprint velocity was strongly related to 100-m sprint time [26]. Besides, the sprint velocity was correlated with spatiotemporal variables in the maximal velocity phase [27]. Based on these points, we decided to analyze 50–60 m interval. To measure sprint times of the 50–60 m interval and of 100 m, reference markers were positioned at 50-, 60-, and 100-m points from the starting line on the right side of the running lane. The 100 m sprint measurements and MR image recordings were separated by 10 ± 18 days (0–61 days).

The mean velocity of the 100 m sprint was calculated by dividing the running distance (100 m) by the 100 m sprint time. The 100 m sprint time was calculated by dividing frame counts from the start to finish of the sprint by the frame rate of the cameras (120 fps). The start of the sprint was defined as the instant when either of the hands were taken off the ground. The finish of the sprint was defined as the instant when the torso overlapped the reference marker at the 100-m point. The mean sprint velocity was also calculated for the 50–60 m interval by dividing the distance of interval (10 m) by the time taken to cover the interval. The sprint time of the interval was calculated by dividing frame counts between the instances when the torso overlapped the reference markers at 50- and 60-m points by the frame rate of the camera (250 fps).

The spatiotemporal variables were calculated in the 50–60 m interval. The stance phase of the right leg was defined as the part of a running cycle from the touchdown of the right foot to the right toe off, and the flight phase was defined from the right toe off to the touchdown of the left foot. The stance and flight times were calculated by dividing each frame count during stance or flight phase by the frame rate of the fixed camera (250 fps). The sum of the stance and flight times was computed as the step time, and its inverse was defined as the step frequency. The stance and flight distances were defined as the horizontal distances that the anterior-posterior center of the pelvis traveled during the corresponding phases, respectively. These horizontal distances were calculated using Image J software (National Institute of Health, USA) by digitizing the anterior-posterior center of the pelvis at the touchdown of the right foot, the right toe off, and the touchdown of the left foot. The sum of the stance and flight distances was computed as the step length. These variables were calculated for two steps within the 50–60 m interval, and the mean values were used for subsequent analyses. The spatiotemporal variables of 10 participants were analyzed twice to evaluate the measurement reproducibility. The CV and ICC for the two measurements were 0.6% and 0.991 for step frequency, 0.7% and 0.983 for step length, 1.4% and 0.960 for flight time, 1.9% and 0.845 for stance time, 1.6% and 0.963 for flight distance, and 1.8% and 0.682 for stance distance, respectively.

### Statistics

Simple linear correlations between two measured variables were tested using a Pearson’s product moment correlation coefficient. According to a previous study [2], it was expected that there would be interactions between the spatiotemporal variables. When such interactions were found, a semi-partial correlation analysis was used to adjust the effect of the other variable. The 95% confidence interval (CI) for the correlations were calculated. Statistical significance was set at *P* < 0.05. All statistical analyses were conducted using IBM SPSS software (version 25; IBM, USA).

## Results

The sprint velocities of 50–60 m interval and of 100 m were 10.2 ± 0.3 m/s and 9.1 ± 0.2 m/s, respectively. There was a significant correlation between sprint velocities of 50–60 m interval and of 100 m (*r* = 0.951, *P* < 0.001). A significant correlation was found between the relative volume of ST and sprint velocity at the 50–60 m interval (*r* = 0.497, *P* = 0.010, Table 1). However, the relative volumes of the other examined muscles were not significantly correlated with the sprint velocity at the 50–60 m interval (*r* = −0.096 to 0.313, *P* = 0.119–0.791).

**Table 1:** Simple correlation coefficients between individual muscle volumes relative to body mass and mean sprint velocity at 50–60 m interval.

Muscle volume	Sprint velocity		
	*r* [95% CI: lower, upper limits]		*P*
GM	0.227	[−0.176, 0.565]	0.265
ST	0.497	[0.136, 0.742]	0.010*
SM	0.244	[−0.158, 0.577]	0.230
BFlh	0.085	[−0.313, 0.457]	0.681
BFsh	−0.096	[−0.466, 0.303]	0.639
ADL	0.064	[−0.332, 0.440]	0.755
ADB	0.055	[−0.340, 0.433]	0.791
ADM	0.313	[−0.085, 0.625]	0.119
Gra	0.187	[−0.216, 0.536]	0.360

* indicates a significant correlation between the muscle volume and sprint velocity (*P* < 0.05).

CI: Confidence interval, GM: Gluteus maximus, ST: Semitendinosus, SM: Semimembranosus, BFlh: Biceps femoris long head, BFsh: Biceps femoris short head, ADL: Adductor longus, ADB: Adductor brevis, ADM: Adductor magnus, Gra: Gracilis.

**Fig 1:**
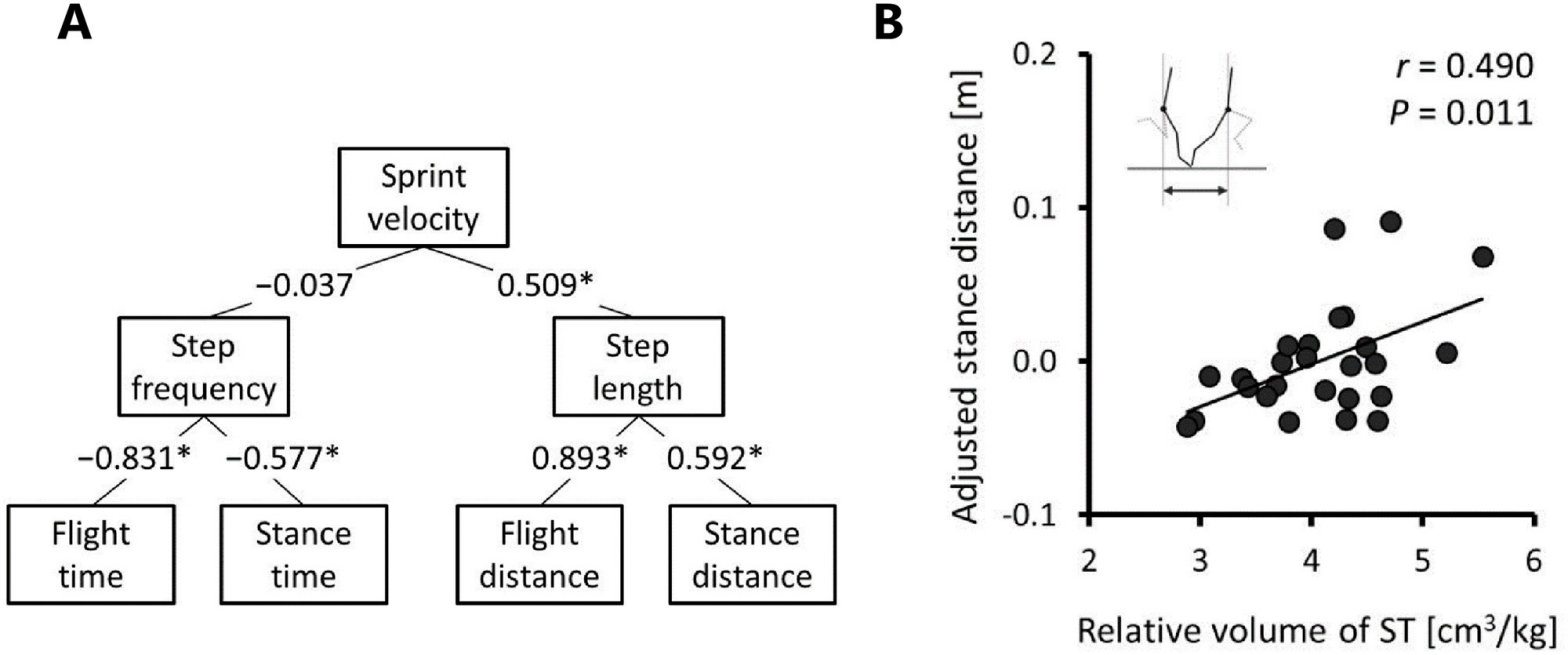
**A**, Simple correlation coefficients between the sprint velocity and the spatiotemporal variables at 50–60 m interval of a 100-m sprint. * indicates *P* < 0.05. The values show the correlation coefficients between the variables. **B**, The relationship between the semitendinosus (ST) volume relative to the body mass and the adjusted stance distance at 50–60 interval of a 100-m sprint. The stance distance was adjusted by the stance time using a semi-partial correlation analysis.

Fig 1A shows correlations between the sprint velocity and the spatiotemporal variables at the 50–60 m interval. Sprint velocity was significantly correlated with step length (*r* = 0.509, *P* = 0.008, Fig 1A), but not with step frequency (*r* = −0.037, *P* = 0.857). Step length was significantly correlated with both the stance distance (*r* = 0.592, *P* = 0.001) and flight distance (*r* = 0.893, *P* < 0.001). The step frequency was significantly correlated with both the stance time (*r* = −0.577, *P* = 0.002) and flight time (*r* = −0.831, *P* < 0.001).

The correlation coefficients among relative volume of ST and the spatiotemporal variables at the 50–60 m interval are presented in Table 2. There were no significant correlations between relative volume of ST and the step frequency (*r* = 0.091, *P* = 0.660) or step length (*r* = 0.166, *P* = 0.418). Among the determinants of the step variables, the relative volume of ST was significantly correlated with stance distance (*r* = 0.414, *P* = 0.036), but not with flight or stance times, or flight distance (*r* = −0.013 to 0.177, *P* = 0.388–0.950). Since a significant correlation was found between the stance distance and stance time (*r* = 0.841, *P* < 0.001), the stance distance was adjusted by the stance time in the semi-partial correlation analysis. The result showed that the relative volume of ST was also significantly correlated with the adjusted stance distance (*r* = 0.490, *P* = 0.011, Fig 1B).

**Table 2:** Simple correlation coefficients between the semitendinosus volume relative to body mass and the spatiotemporal variables at 50–60 m interval.

	Semitendinosus volume		
	*r* [95% CI: lower, upper limits]		*P*
**Step variables**			
Step frequency	−0.095	[−0.465, 0.304]	0.643
Step length	0.278	[−0.123, 0.601]	0.169
**Determinants of step frequency**			
Flight time	−0.013	[−0.398, 0.376]	0.950
Stance time	0.177	[−0.226, 0.528]	0.388
**Determinants of step length**			
Flight distance	0.109	[−0.291, 0.476]	0.597
Stance distance	0.414	[0.032, 0.691]	0.036*

* indicates a significant correlation between the semitendinosus volume and the variable (*P* < 0.05).

CI: Confidence interval.

## Discussion

The present results showed that the relative volume of ST was positively correlated with sprint velocity at the 50–60 m interval, whereas those of the other muscles were not. Furthermore, the relative volume of ST was positively correlated with the stance distance, even after adjustment for the stance time. These results partly support our hypothesis that GM and ST volumes relative to body mass are correlated with spatiotemporal variable(s) and sprint velocity. It has been demonstrated that hip extensor size is related to sprint performance [12–16]. Although we used a cross-sectional design in the present study, the findings suggest that ST size contributes to achieving long stance distance and thereby high sprint velocity.

The sprint velocity at the 50–60 m interval was correlated with step length, but not with step frequency. The step length was correlated with both the stance and flight distances. These results are partially consistent with the results of Hunter et al. [2], who examined the step variables and sprint velocity at the 16 m mark during 25 m sprint. In their study, sprint velocity was correlated only with step length, and step length was correlated with flight distance but not with stance distance. The reasons for these differences are unclear, but may be related to the differences in the sprint phases (maximal velocity vs. acceleration phases) or participants (sprinters vs. athletes who engaged in several types of sporting events). It is likely that in sprinters, long stance distance contributes to achieving long step length and therefore high sprint velocity in the maximal velocity phase.

**Fig 2:**
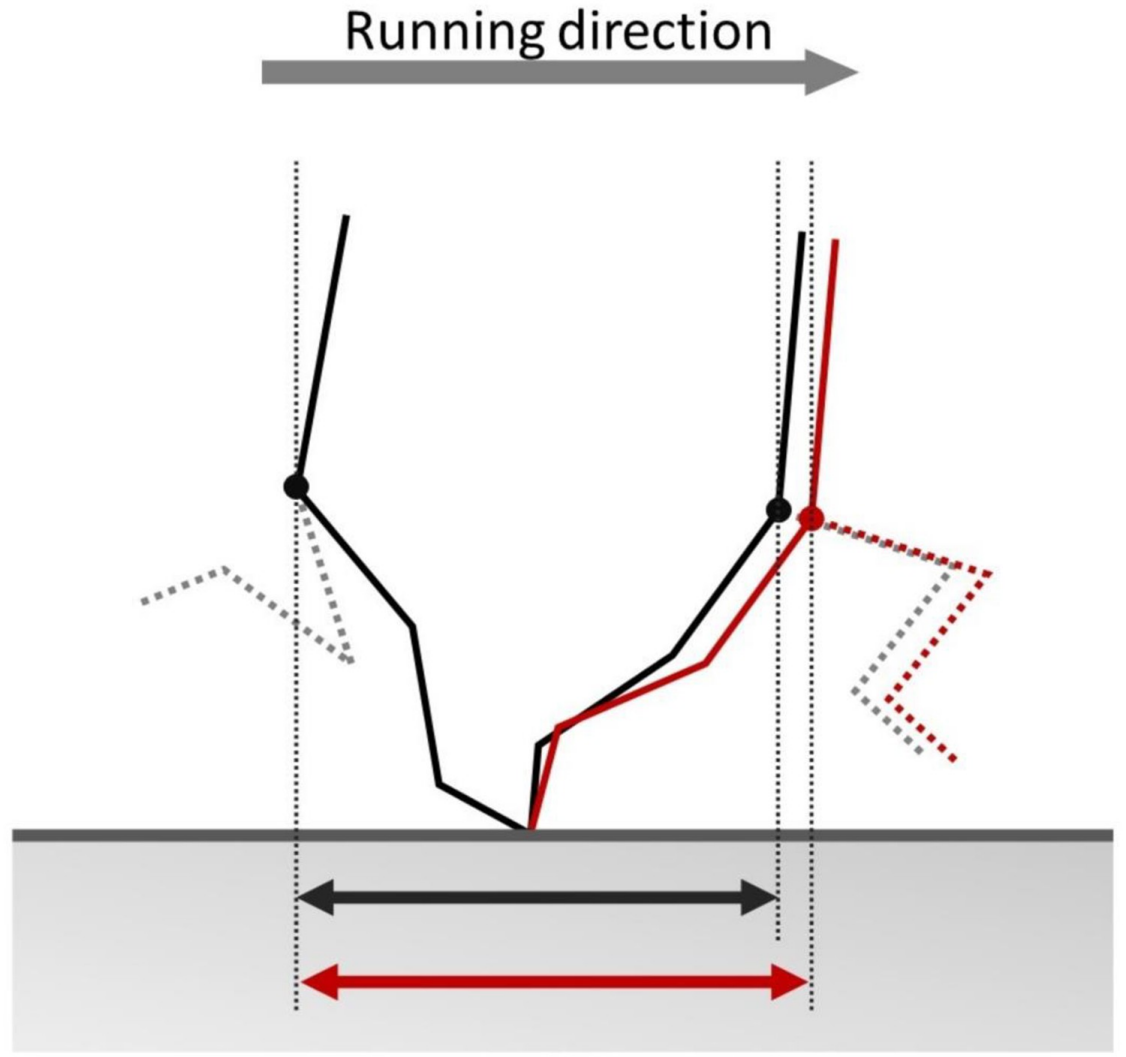
Schematic illustration of the body at touchdown and toe-off (modified from Ito et al. [30]). For a given degree of hip extension and ankle plantar flexion during the stance phase, a small degree of knee extension (red sticks) could result in a longer stance distance compared to a large degree of knee extension (black sticks).

There was a significant correlation between the relative volume of ST and sprint velocity at the 50–60 m interval. In addition, the relative volume of ST was positively correlated with stance distance, even after adjusting for stance time. The semi-partial correlation result suggests that ST contributes to attaining a long stance distance for a given stance time. These results are partly inconsistent with the data of Ema et al. [28], who failed to find a significant correlation between ST volume and sprint velocity at the 35 m mark during 50 m sprint in 15 sprinters. This discrepancy may be due to the differences in sample size (26 vs. 15), shoe conditions (sprint spikes vs. running shoes [R. Ema, personal communication]) and/or the measurement points of sprint velocity (50–60 m interval vs. 35 m). It should be noted that sprint velocities in the present study (10.2 ± 0.3 m/s) were higher than those in the previous study (8.8 ± 0.3 m/s), and presumably reflect the individuals’ maximal sprint velocities [25]. During the maximal velocity phase, the ST was reported to be highly activated from the middle flight to late stance phases in which the hip extension and knee flexion torques were generated [6]. It was speculated that the hip extension and knee flexion torques in the late flight and early stance phases contributed to reducing the horizontal braking force during the stance phase [29], which could lead to an increase in horizontal distance that the center of mass traveled during a limited time of this phase. Therefore, ST can contribute to attaining the long stance distance by generating large hip extension and knee flexion torques during maximal velocity sprinting. Furthermore, the knee flexion torque in the late stance phase can resist the knee extension torque [4], therefore reducing the degree of knee extension. A previous study [30] has suggested that the small degree of knee extension during the stance phase results in a long stance distance for given changes in the hip and ankle joint angles (Fig 2). This may also be a reason for the observed correlation between ST volume and stance distance. Meanwhile, the correlation between ST volume and stance distance was not very strong (*r* = 0.490). This may be related to large inter-individual variability in activity level of ST and BFlh observed during submaximal velocity running [31].

Unlike ST, the relative volumes of the other hamstrings (SM, BFlh, and BFsh) were not significantly correlated with the spatiotemporal variables or sprint velocity. It was reported that the activity level of BFlh was lower than that of ST in the late stance phase [6]. Furthermore, according to a cadaveric study [17], fiber lengths of SM, BFlh, and BFsh are shorter compared to that of ST. Since muscle fiber length is a determinant of the maximal shortening velocity of the muscle [18] and affects its force-velocity relationship (i.e., the muscle with longer fibers can develop a greater force than the muscle with shorter fibers for a given shortening velocity) [19], the short fiber lengths of SM, BFlh, and BFsh are less suited to generating large torques during sprinting where the angular velocity of the hip extensions were reported to be substantially high (e.g., 668 ± 31°/s during stance phase) [20]. Taken together, such inter-synergistic differences in neural activation and morphological properties might account for the differences in the correlation between muscle size and sprint performance. It is likely that the contributions of SM, BFlh, and BFsh to sprint performance are relatively small compared to ST.

There were no significant correlations among the relative volumes of the individual adductors or Gra and the sprint velocity at the 50–60 m interval. These findings are consistent with a previous study [12], in which no significant correlations were found between the volumes of the adductors or Gra and 100 m sprint time. Moreover, volumes of the adductors including the pectineus and Gra were not correlated with sprint velocity at the 35 m mark [28]. However, it was shown that volumes of the adductors, including Gra, were significantly correlated with 40- and 80-m sprint times [15]. Therefore, the associations of volumes of the adductors and Gra with sprint performance remain controversial in the literature. Among the adductors, ADM is the largest muscle [11], and has broad insertions into the femur [32]. For this reason, the moment arm for the hip extension was reportedly different among regions within ADM [33]. Accordingly, the whole volume of ADM measured in the present study included the fibers that contribute less to the hip extension. This might be related to the lack of correlation between the relative volume of ADM and sprint performance.

The relative volume of GM was not significantly correlated with sprint velocity. This result disagrees with previous findings [12,13] that GM volume was negatively correlated with 100 m sprint time. The reasons for the discrepancy between the present and previous results are not clear at this moment, but may be related to the difference in range of participants’ sprint performance: 10.35–11.33 s (present study) vs. 10.23–11.71 s (Sugisaki et al. [12]) and 10.03–11.50 s (Miller et al. [13]). In the previous study [13], GM volume was larger in the elite sprinters (sprint time: 10.03–10.21 s) than in the sub-elite sprinters (sprint time: 10.36–11.50 s). Thus, GM size may be important for achieving shorter sprint time than that of the present participants. In addition, GM also has a broad origin in the pelvis and insertions into the femur [34]. Hence, the contribution of muscle fibers to the hip extension could be variable depending on the regions within GM. Moreover, the activity level during the hip extension exercise was shown to be different among the regions within GM [35]. Therefore, such intramuscular differences in function might account for the lack of significant correlation between whole volume of GM and sprint performance in the present study.

Since large muscles involve a trade-off between high strength capacity and large body mass, it is of importance for athletes and coaches to understand how individual muscle sizes are related to sprint performance. The present study showed that relative volume of ST was associated with maximal sprint velocity and stance distance, whereas the other hamstrings, individual adductors, Gra or GM were not. Although we cannot determine the causality of the relationship based on the cross-sectional observation, the present findings may suggest that a selective increase in ST volume relative to the body mass leads to an increase in stance distance and therefore an increase in sprint velocity. On the other hand, uniform hypertrophy of the individual hip extensors is unlikely to result in an improvement in sprint performance, as the gains in force-generating capacity may not exceed the negative effects of increased mass in most of the muscles. To the best of our knowledge, no study has examined the relationship between changes in ST volume and sprint performance. Bourne et al. [36], demonstrated that knee flexion training, such as Nordic hamstring training induced greater hypertrophy of ST compared to the other hamstrings. Meanwhile, a few studies found an improvement in sprint time after long-term engagement in Nordic hamstring exercise [37,38]. Such training might be beneficial for sprinters whose sprint performances are limited by their short stance distances.

There are several limitations of the present study. First, the present study lacked estimation of muscle activation of the individual hip extensors or joint kinematics and kinetics during sprinting. Further studies incorporating these variables are required to better understand the relationships among the muscle sizes of individual hip extensors and sprint performance. Secondly, the step variables and their determinants were significantly correlated with body height (*r* = −0.720 to 0.636, *P* ≤ 0.019), and therefore, body height can affect the relationships among muscle volumes and spatiotemporal variables. However, the semi-partial correlation analysis that adjusted the stance distance by the stance time also controlled the effect of body height. In fact, there was no significant correlation between the adjusted stance distance and body height (*r* = 0.320, *P* = 0.111). Nonetheless, a significant correlation was found between the adjusted stance distance and relative volume of ST. Thus, the body height may not significantly affect the present findings. Thirdly, nine of the sprinters had a history of strain injury in the hip or thigh muscles during the last 1–4 years prior to this study. It has been indicated that previous hamstring strain injuries are associated with dysfunction of the hamstrings (e.g., low activity level of BFlh during sprinting [39]), which could affect our findings. In this regard, we have performed additional analysis testing the relationship between the relative volume of the individual hip extensors and sprint velocity in the sprinters who had no history of strain injury in the hip or thigh muscles (n = 17). As a result, only relative volume of ST among those of the examined muscles was significantly correlated with sprint velocity of 50–60 m interval (*r* = 0.642, *P* = 0.005, Table A in S2 File). This result was similar to that observed in the original population (n = 26), implying that a history of strain injury may not have a significant influence on the present findings. Lastly, there is a risk of increasing type I error due to the multiple correlation analysis. Meanwhile, the other studies also found a moderate correlation between ST volume normalized to body mass and center of gravity velocity during sprinting (*r* = 0.40, n = 15) [28] or the season best time of 100-m sprint (*r* = −0.34, n = 31) [13], although these correlations were not statistically significant. Therefore, the significant correlations between ST volume, stance distance and sprint velocity in the present study are unlikely to be found only by chance.

In conclusion, the present results demonstrated that the relative ST volume was positively correlated with sprint velocity and stance distance during maximal velocity sprinting. There were no significant correlations among the relative volumes of the other hip extensors and sprint velocity. These findings may suggest that ST size contributes to achieving a long stance distance and thereby a high sprint velocity.

## Supporting information

S1 File Physical characteristics, muscle volumes, sprint velocity, and spatiotemporal variables in each participant. (XLSX)

S2 File Simple correlation coefficients of individual muscle volumes relative to body mass with mean sprint velocity at 50–60 m interval in the sprinters without a history of strain injury in the hip or thigh muscles. (DOCX)

## References

[1] Mero A, Komi PV, Gregor RJ. Biomechanics of sprint running. Sports Med. 1992;13(6):376–92. 10.2165/00007256-199213060-00002 1615256

[2] Hunter JP, Marshall RN, McNair PJ. Interaction of step length and step rate during sprint running. Med Sci Sports Exerc. 2004;36(2):261–71. 10.1249/01.MSS.0000113664.15777.53 14767249

[3] Schache AG, Blanch PD, Dorn TW, Brown NA, Rosemond D, Pandy MG. Effect of running speed on lower limb joint kinetics. Med Sci Sports Exerc. 2011;43(7):1260–71. 10.1249/MSS.0b013e3182084929 21131859

[4] Dorn TW, Schache AG, Pandy MG. Muscular strategy shift in human running: dependence of running speed on hip and ankle muscle performance. J Exp Biol. 2012;215(11):1944–56. 22573774 10.1242/jeb.064527

[5] Wiemann K, Tidow G. Relative activity of hip and knee extensors in sprinting-implications for training. New studies in athletics. 1995;10:29–49.

[6] Higashihara A, Nagano Y, Ono T, Fukubayashi T. Differences in hamstring activation characteristics between the acceleration and maximum-speed phases of sprinting. J Sports Sci. 2018;36(12):1313–8. 10.1080/02640414.2017.1375548 28873030

[7] Hunter JP, Marshall RN, McNair PJ. Segment-interaction analysis of the stance limb in sprint running. J Biomech. 2004;37(9):1439–46. 10.1016/j.jbiomech.2003.12.018 15275853

[8] Morin JB, Gimenez P, Edouard P, et al. Sprint Acceleration Mechanics: The Major Role of Hamstrings in Horizontal Force Production. Front Physiol. 2015;6:404. 10.3389/fphys.2015.00404 26733889 PMC4689850

[9] Ikai M, Fukunaga T. Calculation of muscle strength per unit cross-sectional area of human muscle by means of ultrasonic measurement. Int Z Angew Physiol. 1968;26(1):26–32. 10.1007/BF00696087 5700894

[10] Fukunaga T, Roy R, Shellock F, Hodgson J, Day M, Lee P, et al. Physiological cross-sectional area of human leg muscles based on magnetic resonance imaging. J Orthop Res. 1992;10(6):926–34. 10.1002/jor.1100100623 1403308

[11] Handsfield G, Knaus K, Fiorentino N, Meyer C, Hart J, Blemker S. Adding muscle where you need it: non-uniform hypertrophy patterns in elite sprinters. Scand J Med Sci Sports. 2017;27(10):1050–60. 10.1111/sms.12723 27373796

[12] Sugisaki N, Kobayashi K, Tsuchie H, Kanehisa H. Associations between individual lower-limb muscle volumes and 100-m sprint time in male sprinters. Int J Sports Physiol Perform. 2018;13(2):214–9. 10.1123/ijspp.2016-0703 28605265

[13] Miller R, Balshaw TG, Massey GJ, Maeo S, Lanza MB, Johnston M, et al. The muscle morphology of elite sprint running. Med Sci Sports Exerc. 2020; in press. 10.1249/MSS.0000000000002522 33009196

[14] Sugisaki N, Kanehisa H, Tauchi K, Okazaki S, Iso S, Okada J. The relationship between 30-m sprint running time and muscle cross-sectional areas of the psoas major and lower limb muscles in male college short and middle distance runners. Int J Sport Health Sci. 2011;9:1–7.

[15] Nuell S, Illera-Domínguez VR, Carmona G, Alomar X, Padullés JM, Lloret M, et al. Hypertrophic muscle changes and sprint performance enhancement during a sprint-based training macrocycle in national-level sprinters. Eur J Sport Sci. 2019:1–10. 10.1080/17461391.2019.1668063 31526116

[16] Yasuda T, Kawamoto K, Loenneke JP, Abe T. Magnetic Resonance Imaging-Measured Adductor Muscle Volume and 100 m Sprint Running Performance in Female Sprinters. Int J Clin Med. 2019;10(10):469.

[17] Friederich JA, Brand RA. Muscle fiber architecture in the human lower limb. J Biomech. 1990;23(1):91–5. 10.1016/0021-9290(90)90373-b 2307696

[18] Bodine SC, Roy RR, Meadows DA, Zernicke RF, Sacks RD, Fournier M, et al. Architectural, histochemical, and contractile characteristics of a unique biarticular muscle: the cat semitendinosus. J Neurophysiol. 1982;48(1):192–201. 10.1152/jn.1982.48.1.192 7119845

[19] Lieber RL, Fridén J. Functional and clinical significance of skeletal muscle architecture. Muscle Nerve. 2000;23(11):1647–66. 11054744 10.1002/1097-4598(200011)23:11<1647::aid-mus1>3.0.co;2-m

[20] Nagahara R, Matsubayashi T, Matsuo A, Zushi K. Kinematics of transition during human accelerated sprinting. Biol Open. 2014;3(8):689–99. 10.1242/bio.20148284 24996923 PMC4133722

[21] Faul F, Erdfelder E, Lang AG, Buchner A. G*Power 3: a flexible statistical power analysis program for the social, behavioral, and biomedical sciences. Behav Res Methods. 2007;39(2):175–91. 10.3758/bf03193146 17695343

[22] Chelly SM, Denis C. Leg power and hopping stiffness: relationship with sprint running performance. Med Sci Sports Exerc. 2001;33(2):326–33. 10.1097/00005768-200102000-00024 11224825

[23] Berg H, Tedner B, Tesch P. Changes in lower limb muscle cross-sectional area and tissue fluid volume after transition from standing to supine. Acta Physiol Scand. 1993;148(4):379–85. 10.1111/j.1748-1716.1993.tb09573.x 8213193

[24] Fedorov A, Beichel R, Kalpathy-Cramer J, et al. 3D Slicer as an image computing platform for the Quantitative Imaging Network. Magn Reson Imaging. 2012;30(9):1323–41. 10.1016/j.mri.2012.05.001 22770690 PMC3466397

[25] Ae M, Ito A, Suzuki M. The men’s 100 metres. New studies in athletics. 1992;7(1):47–52.

[26] Slawinski J, Termoz N, Rabita G, Guilhem G, Dorel S, Morin JB, et al. How 100-m event analyses improve our understanding of world-class men’s and women’s sprint performance. Scand J Med Sci Sports, 2017:27(1):45–54. 10.1111/sms.12627 26644061

[27] von Lieres Und Wilkau HC, Bezodis NE, Morin JB, Irwin G, Simpson S, Bezodis IN. The importance of duration and magnitude of force application to sprint performance during the initial acceleration, transition and maximal velocity phases. J Sports Sci, 2020;38(20):2359–2366. 10.1080/02640414.2020.1785193 32627681

[28] Ema R, Sakaguchi M, Kawakami Y. Thigh and psoas major muscularity and its relation to running mechanics in sprinters. Med Sci Sports Exerc. 2018;50(10):2085–91. 10.1249/MSS.0000000000001678 30222688

[29] Mann R, Sprague P. Kinetics of sprinting. In: Proceedings of International Symposium on Biomechanics in Sports; 1983: San Diego (USA). p. 305–16.

[30] Ito A, Ichikawa H, Saito M, Sagawa K, Ito M, Kobayashi K. Relationship between sprint running movement and velocity at full speed phase during a 100 m race. Japan J Phys Educ. 1998;43:260–73. In Japanese.

[31] Hegyi A, Goncalves B, Finni Juutinen T, Cronin N. Individual region-and muscle-specific hamstring activity at different running speeds. Med Sci Sports Exerc. 2019;51(11):2274–2285. 10.1249/MSS.0000000000002060 31634294

[32] Takizawa M, Suzuki D, Ito H, Fujimiya M, Uchiyama E. Why adductor magnus muscle is large: the function based on muscle morphology in cadavers. Scand J Med Sci Sports. 2014;24(1):197–203. 10.1111/j.1600-0838.2012.01466.x 22537037

[33] Dostal WF, Soderberg GL, Andrews JG. Actions of hip muscles. Phys Ther. 1986;66(3):351–9. 10.1093/ptj/66.3.351 3952148

[34] Stern JT. Anatomical and functional specializations of the human gluteus maximus. Am J Phys Anthropol. 1972;36(3):315–39. 10.1002/ajpa.1330360303 4624653

[35] Contreras B, Vigotsky AD, Schoenfeld BJ, Beardsley C, Cronin J. A comparison of two gluteus maximus EMG maximum voluntary isometric contraction positions. PeerJ. 2015;3:e1261. 10.7717/peerj.1261 26417543 PMC4582950

[36] Bourne MN, Duhig SJ, Timmins RG, et al. Impact of the Nordic hamstring and hip extension exercises on hamstring architecture and morphology: implications for injury prevention. Br J Sports Med. 2017;51(5):469–77. 10.1136/bjsports-2016-096130 27660368

[37] Krommes K, Petersen J, Nielsen MB, Aagaard P, Hölmich P, Thorborg K. Sprint and jump performance in elite male soccer players following a 10-week Nordic Hamstring exercise Protocol: a randomised pilot study. BMC Res Notes. 2017;10(1):669. 10.1186/s13104-017-2986-x 29202784 PMC5716363

[38] Ishøi L, Hölmich P, Aagaard P, Thorborg K, Bandholm T, Serner A. Effects of the Nordic Hamstring exercise on sprint capacity in male football players: a randomized controlled trial. J Sports Sci. 2018;36(14):1663–72. 10.1080/02640414.2017.1409609 29192837

[39] Higashihara A, Ono T, Tokutake G, Kuramochi R, Kunita Y, Nagano Y, et al. Hamstring muscles’ function deficit during overground sprinting in track and field athletes with a history of strain injury. J Sports Sci. 2019;37(23):2744–2750. 10.1080/02640414.2019.1664030 31608831

