# Supplementary material for: Muscle size of individual hip extensors in sprint runners: Its relation to spatiotemporal variables and sprint velocity during maximal velocity sprinting
Source: PLoS One. 2021 Apr 5;16(4):e0249670. doi: 10.1371/journal.pone.0249670 (PMC8021153; doi:10.1371/journal.pone.0249670)
Supplement: S2 File — (DOCX) [file pone.0249670.s002.docx]

**Table A. Simple correlation coefficients of individual muscle volumes relative to body mass with mean sprint velocity at 50–60 m interval in the sprinters without a history of strain injury in the hip or thigh muscles (n = 17).**

| Muscle volume | Sprint velocity | | | | |
| --- | --- | --- | --- | --- | --- |
|  |  | *r* [95% CI: lower, upper limits] | | *P* |  |
| GM | 0.479 | | [−0.002, 0.780] | 0.052 | |
| ST | 0.642 | | [0.234, 0.858] | 0.005* | |
| SM | 0.151 | | [−0.356, 0.589] | 0.563 | |
| BFlh | ‒0.132 | | [−0.576, 0.372] | 0.614 | |
| BFsh | 0.099 | | [−0.401, 0.553] | 0.705 | |
| ADL | 0.191 | | [−0.319, 0.615] | 0.463 | |
| ADB | 0.029 | | [−0.458, 0.503] | 0.912 | |
| ADM | 0.236 | | [−0.276, 0.644] | 0.362 | |
| Gra | 0.417 | | [−0.079, 0.748] | 0.095 | |

* indicates a significant correlation between the muscle volume and sprint velocity (*P* < 0.05).

CI: confidence interval, GM: gluteus maximus, ST: semitendinosus, SM: semimembranosus, BFlh: biceps femoris long head, BFsh: biceps femoris short head, ADL: adductor longus, ADB: adductor brevis, ADM: adductor magnus, Gra: gracilis.
